# Identification of priorities for improvement of medication safety in primary care: a PRIORITIZE study

**DOI:** 10.1186/s12875-016-0552-6

**Published:** 2016-11-16

**Authors:** Lorainne Tudor Car, Nikolaos Papachristou, Joseph Gallagher, Rajvinder Samra, Kerri Wazny, Mona El-Khatib, Adrian Bull, Azeem Majeed, Paul Aylin, Rifat Atun, Igor Rudan, Josip Car, Helen Bell, Charles Vincent, Bryony Dean Franklin

**Affiliations:** 1 Department of Primary Care and Public Health, School of Public Health, Imperial College London, London, UK; 2 UCD Conway Institute, gHealth Research Group, The University College Dublin School of Medicine, Dublin, Ireland; 3 Faculty of Health & Social Care, Health & Social Care Programme, The Open University, Milton Keynes, UK; 4 Usher Institute of Population Health Sciences and Informatics, Centre for Global Health Research, The University of Edinburgh Medical School, Edinburgh, UK; 5 Imperial College Health Partners, London, UK; 6 Department of Global Health and Population & Department of Health Policy and Management, Harvard, Boston USA; 7 Health Services and Outcomes Research Programme, LKCMedicine, Nanyang Technological University, Singapore, Singapore; 8 Centre for Medication Safety and Service Quality, Imperial College Healthcare NHS Trust, London, UK; 9 Department of Experimental Psychology, Medical Sciences Division, University of Oxford, Oxford, UK; 10 Centre for Medication Safety and Service Quality, Imperial College Healthcare NHS Trust/UCL School of Pharmacy, London, UK

**Keywords:** Medication error, Patient safety, Priority-setting, Crowd-sourcing, Primary care, Clinicians

## Abstract

**Background:**

Medication error is a frequent, harmful and costly patient safety incident. Research to date has mostly focused on medication errors in hospitals. In this study, we aimed to identify the main causes of, and solutions to, medication error in primary care.

**Methods:**

We used a novel priority-setting method for identifying and ranking patient safety problems and solutions called PRIORITIZE. We invited 500 North West London primary care clinicians to complete an open-ended questionnaire to identify three main problems and solutions relating to medication error in primary care. 113 clinicians submitted responses, which we thematically synthesized into a composite list of 48 distinct problems and 45 solutions. A group of 57 clinicians randomly selected from the initial cohort scored these and an overall ranking was derived. The agreement between the clinicians’ scores was presented using the average expert agreement (AEA). The study was conducted between September 2013 and November 2014.

**Results:**

The top three problems were incomplete reconciliation of medication during patient ‘hand-overs’, inadequate patient education about their medication use and poor discharge summaries. The highest ranked solutions included development of a standardized discharge summary template, reduction of unnecessary prescribing, and minimisation of polypharmacy. Overall, better communication between the healthcare provider and patient, quality assurance approaches during medication prescribing and monitoring, and patient education on how to use their medication were considered the top priorities. The highest ranked suggestions received the strongest agreement among the clinicians, i.e. the highest AEA score.

**Conclusions:**

Clinicians identified a range of suggestions for better medication management, quality assurance procedures and patient education. According to clinicians, medication errors can be largely prevented with feasible and affordable interventions. PRIORITIZE is a new, convenient, systematic, and replicable method, and merits further exploration with a view to becoming a part of a routine preventative patient safety monitoring mechanism.

**Electronic supplementary material:**

The online version of this article (doi:10.1186/s12875-016-0552-6) contains supplementary material, which is available to authorized users.

## Background

Medication errors are preventable mistakes in prescribing, ordering, dispensing, administration and monitoring of drugs that can cause patient harm [1]. Preventable adverse drug events, i.e. injuries that arise from medication errors, are one of the most common and costly patient safety incidents, estimated to affect 2% of adult outpatients and 1.6% of adult inpatients [2]. The estimated financial cost of preventable adverse reactions arising from medication errors amounts each year to more than £770 million in the NHS and $17 billion in the USA [3, 4].

A case note review of prescribing in English primary care detected a 5% prevalence of prescribing or monitoring errors [5]. A systematic review of 13 studies estimated the prevalence of hospital admissions due to medication errors was 3.7% with the majority of incidents judged to be preventable through simple improvements in prescribing [6]. However, evidence on medication error mostly stems from the hospital setting [7, 8]. Evaluating medication safety in primary care is more challenging as administration of medication is largely performed outside the more controlled environment of a healthcare facility [8, 9].

The literature on medication errors is largely retrospective and includes case-note reviews, malpractice data, incident reporting, surveys and direct observation [10–12]. While their frequency is known, it is still unclear which medication errors in primary care are most harmful and should be tackled first. Clinicians’ engagement is essential to determine medication safety priorities and to ensure successful implementation of proactive responses to medication error. A recent study revealed that staff perception of the culture of organisation safety predicted patient safety outcomes [13]. Yet, healthcare providers openly voicing safety concerns may not be part of established healthcare organisational culture [14]. In this study, we used a novel crowd-sourcing, priority-setting methodology to gather and explore clinicians’ views on the causes of, and solutions for, medication errors in primary care.

## Methods

**Fig. 1:**
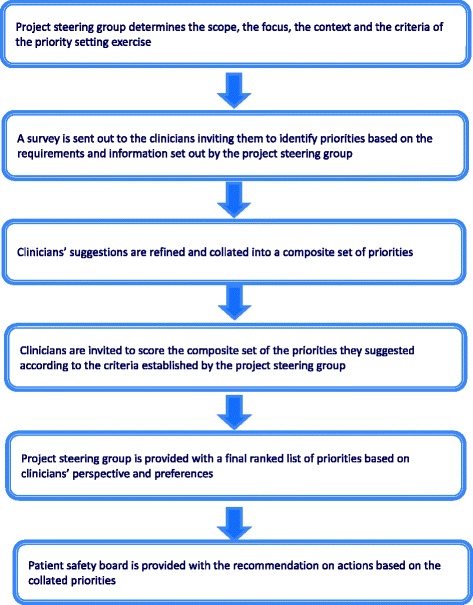
PRIORITIZE methodology flow diagram

In this study, we employed PRIORITIZE, a novel crowd-sourcing, priority-setting methodology (Fig. 1). We developed PRIORITIZE by modifying the Child Health and Nutrition Research Initiative (CHNRI) approach for patient safety context. The CHNRI approach is an established research priority setting methodology and has been used extensively to inform policy makers, funders and international organizations about research gaps and resource priorities [15–17]

In the CHNRI approach, priorities for research are identified and prioritized by international research experts. Correspondingly, in PRIORITIZE, priorities for healthcare services delivery are determined and ranked by clinicians. The PRIORITIZE methodology is designed to determine priorities by simultaneously exploring them from two angles: problems and solutions. The final output of the PRIORITIZE approach is a presentation of the immediate priorities, categorized according to organisational level, for implementation: a) actions for clinicians; b) actions for healthcare organisations; and, c) actions for health system custodians.

This study was deemed to be a service evaluation and quality and safety improvement initiative and consequently did not require ethics approval, research governance approval or informed consent according to the UK’s Health Research Authority guidance [18]. The project steering group (Imperial College Health Partners’ Patient Safety Board) considered previous evidence on patient safety in UK primary care and decided to focus the larger project, of which this study is a part, on medication safety and delayed diagnosis in North West London. This paper describes the findings related to medication safety. North West London has a population of 2.3 million residents, £3.4 billion annual health spend, 440 GP practices employing 1100 GPs and more than 40 000 NHS staff [19]. The patient safety board also determined the criteria for prioritisation of collated suggestions in this study, which were used to score the problems and solutions (Table 1).

**Table 1:** Scoring criteria for prioritization of collated suggestions

For problems
Frequency: This patient safety threat is common
Severity: This patient safety threat leads to high rates of mortality, morbidity and incapacity
Inequity: This patient safety threat affects lower socio-economic groups or ethnic minorities more than other groups
Economic impact: The consequences of this patient safety threat are costly to the healthcare system
Responsiveness to solution: This incident is amenable to a solution within 5 years
For solutions
Feasibility: The implementation of this solution is feasible
Cost-effectiveness: This solution is cost-effective

In the first phase, we developed an open-ended questionnaire for clinicians to identify the main perceived problems and solutions relating to medication safety in primary care. The eligible survey participants were clinicians working in primary care in North West London. It was piloted on a smaller sample of primary care physicians and trainees and underwent multiple rounds of revisions. The final questionnaire (Additional file 1) was distributed in both paper-based and online versions and disseminated via email lists, snowballing (participants were asked to forward the survey to colleagues), and visits to general practices in North West London. We targeted academic and non-academic general practitioners, trainees, pharmacists and nurses.

The collected responses were examined using content analysis with open coding to categorise the free-text responses. Ideas proposed that were sufficiently similar were merged. The coding was performed by one author and subsequently verified by two co-authors. In the second phase, we asked clinicians to score the ideas using four options: score 1 for ‘Yes - I agree with the statement’, score 0 for ‘No - I do not agree with the statement’, score 0.5 for ‘Unsure - I am unsure whether or not I agree’ and blank (no response) for ‘Unaware – I do not feel sufficiently familiar or confident to score this suggestion’ (Additional file 2). This scoring process took about 1 h to complete and we offered a token payment to the participants in a form of a £100 grocery voucher. We invited participants at random from the initial cohort of primary care clinicians to score the priorities. The enrolment for scoring ended after collecting at least 50 completed sheets as per CHNRI guidance (personal communication I. Rudan).

The intermediate scores, i.e. scores for each criterion for every suggestion, were calculated by adding up all the answers (“1,” “0,” or “0.5”) and dividing the sum by the number of received answers. All intermediate scores for all research options were therefore assigned a value between 0 and 100. The overall priority score was then computed as the mean of the scores for each of the five criteria for problems and two criteria for solutions. Higher ranked solutions received more “Yes” responses for each of the criteria and a higher score. The Kappa statistic was deemed an inappropriate test to determine inter-rater agreement in this study due to the sample size, the non-standardized categorical nature of data, the option of blank response to some statements and the number of different criteria used for scoring [20]. Instead, we evaluated inter-rater agreement using the average expert agreement (AEA) [21]. AEA has the ability to expose the suggestions with greatest agreement and controversy as it is shows what proportion of scorers assigned the same score to a particular criterion. AEA does not provide information on statistical significance of any differences between scorers, but it is relevant to policy makers as it gives an indication of the degree of agreement between clinicians in terms of priorities. In calculating AEA, all four possible responses (“Yes”, “No”, “Unsure” and “Unaware”) are treated as a valid response. Therefore, if the substantial proportion of the participants choose “Unaware – I do not feel sufficiently familiar or confident to score this suggestion” as the answer, AEA will reflect this and reduce the level of overall agreement, rather than increase it. The AEA is calculated using the following two formulae for problems and solutions, respectively:

$$ \mathrm{A}\mathrm{E}\mathrm{A} = \kern1em \frac{1}{5}\mathrm{x}{\displaystyle {\sum}_{\mathrm{q}=1}^5\frac{\mathrm{N}\ \left(\mathrm{scorers}\ \mathrm{who}\ \mathrm{provided}\ \mathrm{the}\ \mathrm{most}\ \mathrm{frequent}\ \mathrm{response}\right)}{\mathrm{N}\ \left(\mathrm{scorers}\right)}} $$

$$ \mathrm{A}\mathrm{E}\mathrm{A} = \kern0.5em \frac{1}{3}\mathrm{x}{\displaystyle {\sum}_{\mathrm{q}=1}^3\frac{\mathrm{N}\ \left(\mathrm{scorers}\ \mathrm{who}\ \mathrm{provided}\ \mathrm{the}\ \mathrm{most}\ \mathrm{frequent}\ \mathrm{response}\right)}{\mathrm{N}\ \left(\mathrm{scorers}\right)}} $$

(where q is a question that experts are being asked to evaluate competing patient safety threats (in this case medication errors), ranging from 1 to 5 for problems and 1 to 3 for solutions) [22].

Suggested problems and solutions relating to medication safety in primary care were classified using an adapted model of medication delivery as well as the London Protocol, a framework for analysing risk and safety in clinical practice [23] (Additional file 3).

## Results

**Fig. 2:**
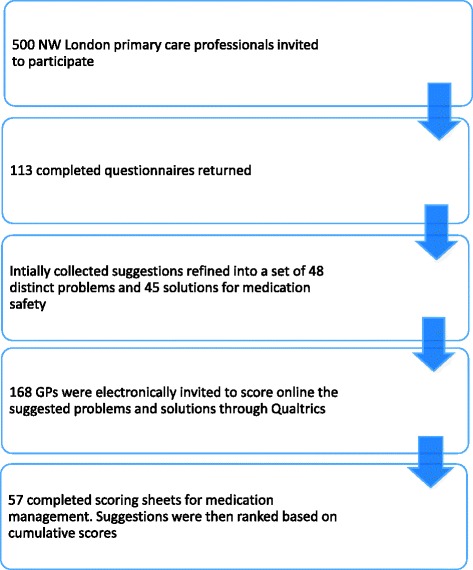
Participants flow diagram

Out of 500 primary care clinicians who were invited to participate, 113 (22.6%) completed the questionnaire. The majority were general practitioners (GPs) (n = 85, 75.2%) (Additional file 4). 175 problems and 147 solutions relating to medication safety in general practice were collected and thematically grouped into a set of 48 distinct problems and 45 solutions for medication safety (Additional file 5: Table S5 and Additional file 6: Table S6). From the initial primary care clinicians’ cohort, 168 GPs were invited to score the composite list of suggestions resulting in 57 fully completed scoring sheets (Fig. 2). Collated problems and solutions were ranked based on the scores obtained.

The three top ranked problems leading to medication errors were incomplete reconciliation of medication during patient ‘hand-overs’, inadequate patient education about how to take their medications and poor discharge summaries (Table 2). The three highest ranked solutions were standardized discharge summary templates, reduction of unnecessary prescribing, and avoidance of polypharmacy (Table 3).

**Table 2:** Top ten medication related problems in primary care^a^

RANK	Highlighted medication related problems in primary care	Total priority score	Breakdown point in the medication process	Contributory factor
1	Incomplete reconciliation of medication during patient ‘hand-overs’ such as admission to and discharge from hospital or emergency department	86.7	Transfer of care	Individual staff
2	Incorrect or insufficient patient education about the use of their medication e.g. how to take bisphosphonate or an inhaler	86.3	Administering	Patient
3	Poor discharge summaries	83.8	Transfer of care	Individual staff
4	Polypharmacy in the elderly	81.3	Monitoring	Patient
5	Patient’s inability to understand or remember information about the daily doses or time of administration	81.3	Administering	Patient
6	Repeat prescribing without proper review, leading to continued use of unnecessary or unsafe medications	80.6	Monitoring	Individual staff
7	Time pressures leading to prescribing errors and extended medication review times	79.2	Prescribing	Work environment
8	Long-term prescribing of non-steroidal anti-inflammatory drugs without reviewing if there is an ongoing need for them	77.9	Prescribing	Individual staff
9	Repeat prescribing of pain-killers including opiates without a regular review of true need or alternatives	77.9	Prescribing	Individual staff
10	Delays in receiving notes when patient changes the practice they are registered with	77.3	Transfer of care	Task design

(Clinicians scored problems using the following criteria: frequency, severity, inequity, economic impact and responsiveness to solution (Table 1). The scoring options were 1 for “yes (e.g. this problem is common)”, 0 for “no (e.g. this problem is uncommon)”, 0.5 for “unsure (e.g. I am unsure if this problem is common)” and blank for “unaware (e.g. I do not know if this problem is common)”. Total Priority score is the mean of the scores for each of the five criteria and could range from 0 to 100. Higher ranked problems received more “Yes” responses for each of the criteria and a higher score)

^a^All tables use clinicians’ verbatim statements which were only exceptionally reworded for clarity

**Table 3:** Top twelve solutions for medication safety in primary care

RANK	Proposed solution for medication safety problems in primary care	Total priority score	Breakdown point	Type of solution
1	Develop standardized template for discharge summaries (e.g. with clear indications of changes from admission to discharge and with rationale)	97.4	Prescribing	Task design
2	Reduce unnecessary medication/antibiotic prescribing	96.4	Prescribing	Task design
3	Minimize polypharmacy	94.3	Administering	Task design
4	Take patient’s co-morbidities more carefully into consideration when prescribing medications	93.8	Prescribing	Individual staff
5	To give clear guidelines to patients as to how frequently they need medication reviews	92.7	Communication with patient/carers	Patient
6	Computer system to automatically inform the patient and the GP when blood tests are overdue	92.7	Monitoring	Task design
7	Increase the use of e-discharge letters	91.7	Prescribing	Task design
8	Improve patient information leaflets	91.1	Communication with patient/carers	Patient
9	Unified medication and investigations records that have listings of allergies and current medications of patients across primary and secondary care. This will allow GPs to see who changed, why, when and what medication or a result of a test ordered in secondary care	90.6	Transfer of care	Task design
10	The development of shared care protocols	90.1	Transfer of care	Task design
11	Pharmacies should offer a check-and-collect service in addition to dispensing to only supply what’s needed. Explanation: Patients often have cupboards full of old medications	90.1	Dispensing	Task design
12	To write indications next to each prescribed medication	90.1	Prescribing	Task design

(Clinicians scored solutions using the following criteria: feasibility and cost-effectiveness (Table 1). The scoring options were 1 for “yes (e.g. this solution is feasible)”, 0 for “no (e.g. this solution is unfeasible)”, 0.5 for “unsure (e.g. I am unsure if this solution is feasible)” and blank for “unaware (e.g. I do not know if this solution is feasible)”. Total Priority score is the mean of the scores for each of the three criteria and ranges from 0 to 100. Higher ranked solutions received more “Yes” responses for each of the criteria and a higher score)

Overall, clinicians identified monitoring and prescribing as the medication stages most vulnerable to medication errors. However, the top 5 problems mostly referred to routine matters such as errors during transfer of care and administering of medication. Poor care coordination and communication between different services and providers of care, lack of or inappropriate medication reviews and monitoring as well as patient-related factors (e.g. polypharmacy or memory issues) were seen as the main medication safety concerns (Additional file 5: Table S5). Several problems focused on inappropriate prescribing in specific circumstances e.g. for pain management, antipsychotics, teratogenic medications in pregnancy, for example.

Many of the suggested solutions focused on re-design of medication delivery, e.g. by developing standardized discharge summaries, unified investigation and medication records, shared care protocols or patient-held records. Information technology was also considered important for the streamlining of care and improvement of safety (e.g. use of e-discharge letters and e-referrals, computer system alerts for overdue blood tests and warnings when re-issuing medication). The most cost-effective solutions according to clinicians were minimising polypharmacy, taking patients’ comorbidities into consideration when prescribing and introducing standardized discharge summaries. The most feasible solutions according to clinicians were standardized discharge summaries, healthcare assistants’ home visits to ensure medication adherence, and unified medication and investigations records that have listings of allergies and current medications of patients across primary and secondary care (Additional file 6: Table S6).

The top ranked solutions corresponded to the top ranked problems. The highest ranked suggestions had the highest AEA, i.e. there was a stronger consensus among clinicians for the top suggestions compared to those ranked lower. Proposed solutions received higher AEA scores compared to problems, i.e. clinicians agreed more on the ranking of solutions compared to the ranking of problems (Additional file 5: Table S5).

## Discussion

In this study, clinicians identified priorities for improving medication safety in primary care. The top three problems were incomplete reconciliation of medication during patient ‘hand-overs’, inadequate patient education about their medication use and poor discharge summaries. The highest ranked solutions included development of a standardized discharge summary template, reduction of unnecessary prescribing, and minimization of polypharmacy. Overall, improving communication among clinicians and with patients, re-design of medication delivery and patient education were seen as key areas for improvement of medication safety. Many identified suggestions in our study are feasible, affordable and could contribute to improvements to medication safety in primary care. Some would, however, require more time within already time-pressured clinical encounters.

There is a paucity of research on underlying causes of, and solutions for, medication errors in the primary care setting [5, 24]. The existing evidence stems mostly from secondary care and addresses certain stages of medication delivery rather than the whole process [25]. This fragmented approach prevents a systems-oriented perspective to the minimisation of medication errors. Our study encompasses all steps in medication delivery in primary care, tackling both the contributory factors and the prevention strategies. Such dual strategy was useful since many of the suggested problems focused on individual failure, whereas the proposed solutions focused on organisational re-design.

Clinicians in our study perceived prescribing and monitoring of medication as particularly susceptible to safety incidents. Similarly, a systematic review on medication safety in primary care showed that most medication errors occur during prescribing and monitoring stages [25]. However, the top identified problems mostly focus on routine, service delivery-related matters rather than areas requiring clinical decision making skills, e.g. incomplete reconciliation of medication, repeat prescribing without proper review or delays in receiving patient notes. Notably, clinicians in our study, instead of addressing difficulties with prescribing, prioritised much more mundane and fixable problems.

Poor communication among clinicians and with patients as well as incomplete healthcare documentation were seen as the main safety threats which reinforces findings from previous work [26, 27]. Research shows that direct communication between hospital and primary care physicians is often sporadic and accompanied by discharge summaries missing important drug-related information [27–29]. As a possible solution, our study suggested that a standardized discharge summary template clearly indicating changes in medication may improve information transfer and medication safety. Recent studies show that the use of templates can improve the quality of discharge summaries [30, 31]

Lack of medication adherence is considered one of the main patient-related medication safety threats in primary care [25]. In our study, several suggestions focused on ensuring medication adherence through patient education and support. Correspondingly, patient education was also identified by the international patient safety experts as a safety imperative [9, 32, 33]. A Cochrane review showed that provision of patient self-monitoring and self-management programmes improved medication safety [34]. Our study identified discrete methods of educating and empowering patients to be more involved in their healthcare; namely, giving clear guidelines to patient about how frequently they require medication reviews and using computer systems to inform the patient about overdue blood tests.

A number of identified solutions to medication errors in our study focused on organisational changes and re-design of particular medication delivery tasks. It is unclear how effective these strategies are as the literature on effective interventions to reduce medication errors is lacking in terms of the experimental study designs, breadth of evaluated interventions and types of outcomes. Among the organisation-level solutions identified in our study, the use of clinical computer systems (in the form of e-discharge letters, e-referrals, alerts etc.) was perceived beneficial which corresponds to the evidence showing that the use of information technology prevents medication errors [35–39].

### Strengths and limitations

PRIORITIZE is a modified version of an established priority-setting methodology [15–17]. It is transparent, systematic, easily reproducible and anonymous. PRIORITIZE enables open and blame-free expression of safety concerns, recommendations and ideas from many participants. Potential limitations of this study relate to the generalizability and validity of the findings. There is a possibility of selection bias as a self-selected sample was recruited and the respondents in our study potentially differed from the non-respondents. All invited participants had the same eligibility criteria by being a primary care healthcare provider in North West London but there may have been other, unmeasured biases such as clinicians with specific views being more likely to respond. The findings are perhaps not generalizable to other healthcare settings or systems, which have a different organization of primary care. However, they correspond to the available international literature and should be seen as pertinent beyond the study setting. The modest response rate in this study matches other clinician surveys and evidence showing that physicians often decline to participate in surveys [40, 41]. Longer, online surveys and those with open-ended questions (such as our survey) are particularly prone to a poor response rate [42]. Increasing participation and improving reliability of findings could be achieved by introducing PRIORITIZE as a standard part of the organizational quality improvement efforts.

The PRIORITIZE approach is at an early stage and could be further improved. For example, it may be useful to provide examples that would guide the specificity and type of the suggestions (e.g. error producing conditions, errors and adverse events) or to ensure longitudinal data collection through repeated annual questionnaires. Different types of analyses could be applied, e.g. determining the suitable healthcare implementation level for each solution, choosing prioritization criteria most important to the healthcare organisation (e.g. urgency, impact, affordability, execution risk, sustainability etc.), analysing correlation between priorities, analysing patterns of priorities for different staff groups or regional areas, or undertaking an in-depth comparison of providers’ and patients’ views.

## Conclusion

Medication errors in primary care are common, impactful and yet under-investigated. In our study, clinicians identified a range of areas of medication safety that were potentially amenable to improvement as well as discrete interventions that were achievable. Better care coordination, robust quality assurance mechanisms and improved patient education were seen as key to the prevention of medication errors in primary care. This bottom-up approach (in which staff views drive change) is essential for successful implementation of new healthcare policies. This study offers unique value in that suggestions were in line with health system custodians’ identified needs. Its findings are being used to guide Imperial College Health Partners’ work on Medicines Optimisation in North West London. Their Patient Safety Board determined the priorities that were synergistic or inter-related (e.g. “minimising polypharmacy”, “reduction of unnecessary antibiotic prescribing”, “long-term prescribing of nonsteroidal anti-inflammatory drugs (NSAIDs) without reviewing if there is an on-going need for it”, “repeat prescribing of pain-killers including opiates without a regular review of true need or alternatives”) to address them with a focused and concerted effort. This association among identified suggestions reaffirmed the importance of certain priorities and gave a clear message where action is needed. Next steps should include synthesis of the existing evidence relating to the identified suggestions to determine effective interventions.

This novel priority setting approach offers many advantages to healthcare policy makers. PRIORITIZE corresponds to the recent policy decisions to involve healthcare staff in patient safety research and is a complementary method to current exploratory tools used for mapping of primary care safety priorities [7, 43, 44]. Following further validation of the method, it may be used as a routine mechanism for staff feedback to identify safety threats at different healthcare levels, increase patient safety awareness and improve organisational culture.

## Additional files

Additional file 1: Initial questionnaire on problems and solutions related to medication safety and delayed diagnosis in primary care. (ODT 60 kb)
Additional file 2: Scoring questionnaire. (ODT 136 kb)
Additional file 3: Framework used for analysis of Contributing Factors to medication safety in primary care (based on the London protocol). (ODT 5 kb)
Additional file 4: Characteristics of the respondents to the initial questionnaire. (ODT 6 kb)
Additional file 5: Table S5. Ranking of all (48) medication-related problems from primary care clinicians’ perspective (AEA range: 0 to 1) (ODT 20 kb)
Additional file 6: Table S6. Ranking of all (45) solutions to medication-related problems from primary care clinicians’ perspective (AEA range: 0 to 1). (ODT 17 kb)

## Abbreviations

AEA: Average experts’ agreement
GP: General practitioners
