# Supplementary material for: Identification of priorities for improvement of medication safety in primary care: a PRIORITIZE study
Source: BMC Fam Pract. 2016 Nov 16;17:160. doi: 10.1186/s12875-016-0552-6 (PMC5112691; doi:10.1186/s12875-016-0552-6)
Supplement: Supplementary file 2 [file 12875_2016_552_MOESM2_ESM.odt]

Supplementary information S2 Text. Scoring questionnaire

| https://imperial.eu.qualtrics.com/CP/Graphic.php?IM=IM_b2BEPWfJGjjJsCF |
| --- |
| Patient safety priorities in general practice   Dear Colleague,   Thank you for participating in the first step of this process where you identified patient safety problems in general practice and proposed solutions. They are all listed below. In this second and final step, please indicate your agreement or disagreement with all the statements in the table below using the following criteria:  Y(es) : if you agree N(o) : if you disagree UnS(ure) : if you are aware of the problem, but unsure about the answer UnA(ware) : if you are not sufficiently aware of the problem |
|  |
|  |
| Medication-related patient safety problems in general practice | This patient safety threat is common | This patient safety threat leads to high rates of mortality, morbidity and incapacity | This patient safety threat affects more lower socio-economic groups or ethnic minorities | The consequences of this patient safety threat are costly to the healthcare system | This incident is amenable to a solution within 5 years |
|  | Y | N | UnS | UnA | Y | N | UnS | UnA | Y | N | UnS | UnA | Y | N | UnS | UnA | Y | N | UnS | UnA |
| 01. Patient taking the wrong dose | ○ | ○ | ○ | ○ | ○ | ○ | ○ | ○ | ○ | ○ | ○ | ○ | ○ | ○ | ○ | ○ | ○ | ○ | ○ | ○ |
| 02. Illegible specialist’s prescription | ○ | ○ | ○ | ○ | ○ | ○ | ○ | ○ | ○ | ○ | ○ | ○ | ○ | ○ | ○ | ○ | ○ | ○ | ○ | ○ |
| 03. The history of past patient safety threats and adverse reactions to medications (e.g. NSAIDs and allergies) are not recorded in the patient notes (coding issues) | ○ | ○ | ○ | ○ | ○ | ○ | ○ | ○ | ○ | ○ | ○ | ○ | ○ | ○ | ○ | ○ | ○ | ○ | ○ | ○ |
| 04. Continued pressure to change to cheapest medications | ○ | ○ | ○ | ○ | ○ | ○ | ○ | ○ | ○ | ○ | ○ | ○ | ○ | ○ | ○ | ○ | ○ | ○ | ○ | ○ |
| 05. Switching between generics and branded medication e.g. for antiepileptic medication | ○ | ○ | ○ | ○ | ○ | ○ | ○ | ○ | ○ | ○ | ○ | ○ | ○ | ○ | ○ | ○ | ○ | ○ | ○ | ○ |
| 06. Patient being issued repeat prescriptions for an ACE-inhibitor without regular checks of U+Es | ○ | ○ | ○ | ○ | ○ | ○ | ○ | ○ | ○ | ○ | ○ | ○ | ○ | ○ | ○ | ○ | ○ | ○ | ○ | ○ |
| 07. Incorrect or insufficient patient education about the use of their medication e.g. how to take biphosphate or an inhaler | ○ | ○ | ○ | ○ | ○ | ○ | ○ | ○ | ○ | ○ | ○ | ○ | ○ | ○ | ○ | ○ | ○ | ○ | ○ | ○ |
| 08. Medication has not been taken by a patient due to cost | ○ | ○ | ○ | ○ | ○ | ○ | ○ | ○ | ○ | ○ | ○ | ○ | ○ | ○ | ○ | ○ | ○ | ○ | ○ | ○ |
| 09. Nationals of other countries keep the GP in their country of origin and ask for medication that has been prescribed abroad without full information regarding indication, dose, etc. This leads to the wrong medication prescribed inadvertently | ○ | ○ | ○ | ○ | ○ | ○ | ○ | ○ | ○ | ○ | ○ | ○ | ○ | ○ | ○ | ○ | ○ | ○ | ○ | ○ |
| Medication-related patient safety problems in general practice | This patient safety threat is common | This patient safety threat leads to high rates of mortality, morbidity and incapacity | This patient safety threat affects more lower socio-economic groups or ethnic minorities | The consequences of this patient safety threat are costly to the healthcare system | This incident is amenable to a solution within 5 years |
|  | Y | N | UnS | UnA | Y | N | UnS | UnA | Y | N | UnS | UnA | Y | N | UnS | UnA | Y | N | UnS | UnA |
| 10. Clicking/selecting the wrong drug/dose in the drop-down menu | ○ | ○ | ○ | ○ | ○ | ○ | ○ | ○ | ○ | ○ | ○ | ○ | ○ | ○ | ○ | ○ | ○ | ○ | ○ | ○ |
| 11. Repeat prescribing without proper review, leading to continued use of unnecessary or unsafe medications | ○ | ○ | ○ | ○ | ○ | ○ | ○ | ○ | ○ | ○ | ○ | ○ | ○ | ○ | ○ | ○ | ○ | ○ | ○ | ○ |
| 12. Insufficient checking of blood tests of patients on medications that require regular monitoring e.g. methotrexate, sulphasalazine, isotretinoin, lithium, etc. | ○ | ○ | ○ | ○ | ○ | ○ | ○ | ○ | ○ | ○ | ○ | ○ | ○ | ○ | ○ | ○ | ○ | ○ | ○ | ○ |
| 13. Confusion in medication names leading to patient safety threats | ○ | ○ | ○ | ○ | ○ | ○ | ○ | ○ | ○ | ○ | ○ | ○ | ○ | ○ | ○ | ○ | ○ | ○ | ○ | ○ |
| 14. The “SystmOne” repeat prescriptions feature can allow patients to potentially get too much medication | ○ | ○ | ○ | ○ | ○ | ○ | ○ | ○ | ○ | ○ | ○ | ○ | ○ | ○ | ○ | ○ | ○ | ○ | ○ | ○ |
| 15. Incomplete reconciliation of medication during patient ‘hand-overs’ such as admission to and discharge from hospital or A&E | ○ | ○ | ○ | ○ | ○ | ○ | ○ | ○ | ○ | ○ | ○ | ○ | ○ | ○ | ○ | ○ | ○ | ○ | ○ | ○ |
| 16. Poor discharge summaries | ○ | ○ | ○ | ○ | ○ | ○ | ○ | ○ | ○ | ○ | ○ | ○ | ○ | ○ | ○ | ○ | ○ | ○ | ○ | ○ |
| 17. Delays in receiving notes when patient changes the practice they are registered with | ○ | ○ | ○ | ○ | ○ | ○ | ○ | ○ | ○ | ○ | ○ | ○ | ○ | ○ | ○ | ○ | ○ | ○ | ○ | ○ |
| 18. Pharmacies ordering prescriptions for patients early and then omitting any changes | ○ | ○ | ○ | ○ | ○ | ○ | ○ | ○ | ○ | ○ | ○ | ○ | ○ | ○ | ○ | ○ | ○ | ○ | ○ | ○ |
| 19. Insufficient monitoring of INR in patients on Warfarin | ○ | ○ | ○ | ○ | ○ | ○ | ○ | ○ | ○ | ○ | ○ | ○ | ○ | ○ | ○ | ○ | ○ | ○ | ○ | ○ |
| 20. Some medications being prescribed in dosette boxes and others are not | ○ | ○ | ○ | ○ | ○ | ○ | ○ | ○ | ○ | ○ | ○ | ○ | ○ | ○ | ○ | ○ | ○ | ○ | ○ | ○ |
| 21. Computer problems: the patient file that is being opened is not that of the patient who is in the room at the time of the consultation. This can happen with EMIS Web | ○ | ○ | ○ | ○ | ○ | ○ | ○ | ○ | ○ | ○ | ○ | ○ | ○ | ○ | ○ | ○ | ○ | ○ | ○ | ○ |
| 22. The “SystmOne” screen feels more prone to errors as it is very full, the alerts are less concise and the GP does not have to tick a box before issuing medication | ○ | ○ | ○ | ○ | ○ | ○ | ○ | ○ | ○ | ○ | ○ | ○ | ○ | ○ | ○ | ○ | ○ | ○ | ○ | ○ |
| 23. Too many irrelevant pop-ups and warnings on the computer screen (e.g. for interactions) which lead to the relevant items being ignored | ○ | ○ | ○ | ○ | ○ | ○ | ○ | ○ | ○ | ○ | ○ | ○ | ○ | ○ | ○ | ○ | ○ | ○ | ○ | ○ |
| 24. Polypharmacy in the elderly | ○ | ○ | ○ | ○ | ○ | ○ | ○ | ○ | ○ | ○ | ○ | ○ | ○ | ○ | ○ | ○ | ○ | ○ | ○ | ○ |
| 25. Limited continuity of care i.e. numerous doctors involved in the care of the same patient can lead to poor knowledge of the patient and the wrong medication being prescribed to them | ○ | ○ | ○ | ○ | ○ | ○ | ○ | ○ | ○ | ○ | ○ | ○ | ○ | ○ | ○ | ○ | ○ | ○ | ○ | ○ |
| 26. Long-term prescribing of NSAIDs without reviewing if there is an ongoing need for it | ○ | ○ | ○ | ○ | ○ | ○ | ○ | ○ | ○ | ○ | ○ | ○ | ○ | ○ | ○ | ○ | ○ | ○ | ○ | ○ |
| 27. Repeat prescribing of pain-killers including opiates without a regular review of true need or alternatives | ○ | ○ | ○ | ○ | ○ | ○ | ○ | ○ | ○ | ○ | ○ | ○ | ○ | ○ | ○ | ○ | ○ | ○ | ○ | ○ |
| 28. Wrong dose calculations for children and patients with renal disease and in other special situations | ○ | ○ | ○ | ○ | ○ | ○ | ○ | ○ | ○ | ○ | ○ | ○ | ○ | ○ | ○ | ○ | ○ | ○ | ○ | ○ |
| 29. GP advised by a specialist or hospital to prescribe a new medication without being given guidance on how to monitor this medication | ○ | ○ | ○ | ○ | ○ | ○ | ○ | ○ | ○ | ○ | ○ | ○ | ○ | ○ | ○ | ○ | ○ | ○ | ○ | ○ |
| 30. Patient falls after being given medication (e.g. tranquilizers) | ○ | ○ | ○ | ○ | ○ | ○ | ○ | ○ | ○ | ○ | ○ | ○ | ○ | ○ | ○ | ○ | ○ | ○ | ○ | ○ |
| Medication-related patient safety problems in general practice | This patient safety threat is common | This patient safety threat leads to high rates of mortality, morbidity and incapacity | This patient safety threat affects more lower socio-economic groups or ethnic minorities | The consequences of this patient safety threat are costly to the healthcare system | This incident is amenable to a solution within 5 years |
|  | Y | N | UnS | UnA | Y | N | UnS | UnA | Y | N | UnS | UnA | Y | N | UnS | UnA | Y | N | UnS | UnA |
| 31. Hypotension and postural hypotension from antihypertensive | ○ | ○ | ○ | ○ | ○ | ○ | ○ | ○ | ○ | ○ | ○ | ○ | ○ | ○ | ○ | ○ | ○ | ○ | ○ | ○ |
| 32. Hypocalcaemia in chronic renal failure | ○ | ○ | ○ | ○ | ○ | ○ | ○ | ○ | ○ | ○ | ○ | ○ | ○ | ○ | ○ | ○ | ○ | ○ | ○ | ○ |
| 33. Hypoglycaemia due to taking medicines for diabetes | ○ | ○ | ○ | ○ | ○ | ○ | ○ | ○ | ○ | ○ | ○ | ○ | ○ | ○ | ○ | ○ | ○ | ○ | ○ | ○ |
| 34. Lack of clarity about the drugs that are to be tailed-off following a discharge (e.g. steroids) | ○ | ○ | ○ | ○ | ○ | ○ | ○ | ○ | ○ | ○ | ○ | ○ | ○ | ○ | ○ | ○ | ○ | ○ | ○ | ○ |
| 35. Prescribing of benzodiazepines when other alternatives exist | ○ | ○ | ○ | ○ | ○ | ○ | ○ | ○ | ○ | ○ | ○ | ○ | ○ | ○ | ○ | ○ | ○ | ○ | ○ | ○ |
| 36. Prescribing of rare, unusual and new drugs that the GP is not skilled in their use | ○ | ○ | ○ | ○ | ○ | ○ | ○ | ○ | ○ | ○ | ○ | ○ | ○ | ○ | ○ | ○ | ○ | ○ | ○ | ○ |
| 37. Prescribing teratogen medications during pregnancy | ○ | ○ | ○ | ○ | ○ | ○ | ○ | ○ | ○ | ○ | ○ | ○ | ○ | ○ | ○ | ○ | ○ | ○ | ○ | ○ |
| 38. Time pressures- leading to prescribing errors and extended medication review times | ○ | ○ | ○ | ○ | ○ | ○ | ○ | ○ | ○ | ○ | ○ | ○ | ○ | ○ | ○ | ○ | ○ | ○ | ○ | ○ |
| 39. Incomplete coding of patient problems | ○ | ○ | ○ | ○ | ○ | ○ | ○ | ○ | ○ | ○ | ○ | ○ | ○ | ○ | ○ | ○ | ○ | ○ | ○ | ○ |
| 40. Overdose, underdose or taking the wrong medications due to memory issues leading to a deterioration of health status | ○ | ○ | ○ | ○ | ○ | ○ | ○ | ○ | ○ | ○ | ○ | ○ | ○ | ○ | ○ | ○ | ○ | ○ | ○ | ○ |
| 41. Patient’s inability to understand or remember information about the daily doses or time of administration | ○ | ○ | ○ | ○ | ○ | ○ | ○ | ○ | ○ | ○ | ○ | ○ | ○ | ○ | ○ | ○ | ○ | ○ | ○ | ○ |
| 42. Patient’s inability to articulate or recognize medication side effects that need action | ○ | ○ | ○ | ○ | ○ | ○ | ○ | ○ | ○ | ○ | ○ | ○ | ○ | ○ | ○ | ○ | ○ | ○ | ○ | ○ |
| 43. Carers not recognising medication side effects | ○ | ○ | ○ | ○ | ○ | ○ | ○ | ○ | ○ | ○ | ○ | ○ | ○ | ○ | ○ | ○ | ○ | ○ | ○ | ○ |
| 44. Carers allowed only to prompt and not give medication | ○ | ○ | ○ | ○ | ○ | ○ | ○ | ○ | ○ | ○ | ○ | ○ | ○ | ○ | ○ | ○ | ○ | ○ | ○ | ○ |
| 45. Lack of medication review in home bound patients | ○ | ○ | ○ | ○ | ○ | ○ | ○ | ○ | ○ | ○ | ○ | ○ | ○ | ○ | ○ | ○ | ○ | ○ | ○ | ○ |
| 46. Inappropriate prescribing of antipsychotics due to lack of clarity around indications for people with dementia leading to a deterioration of patient’s health or increased confusion | ○ | ○ | ○ | ○ | ○ | ○ | ○ | ○ | ○ | ○ | ○ | ○ | ○ | ○ | ○ | ○ | ○ | ○ | ○ | ○ |
| 47. Treatment of anger, aggression and violence with medication which is not licenced for these indications in elderly leading to drowsiness or even coma/death | ○ | ○ | ○ | ○ | ○ | ○ | ○ | ○ | ○ | ○ | ○ | ○ | ○ | ○ | ○ | ○ | ○ | ○ | ○ | ○ |
| 48. Inappropriate prescribing of pain management medication leading to drowsiness, falls | ○ | ○ | ○ | ○ | ○ | ○ | ○ | ○ | ○ | ○ | ○ | ○ | ○ | ○ | ○ | ○ | ○ | ○ | ○ | ○ |
| Please add any further ideas or comments that you believe are important and should be included in this study. |
|  |  |
|  |  |  |  |  |  |  |  |  |  |  |  |  |  |  |  |  |  |  |  |  |
| Y(es) : if you agree N(o) : if you disagree UnS(ure) : if you are aware of the problem, but unsure about the answer UnA(ware) : if you are not sufficiently aware of the problem |
| Suggested solutions that could reduce or prevent medication-related patient safety problems in general practice | This solution is cost-effective | The implementation of this solution is feasible |
| Y | N | UnS | UnA | Y | N | UnS | UnA |
| 01. Continuously and systematically double-check medication changes | ○ | ○ | ○ | ○ | ○ | ○ | ○ | ○ |
| 02. Closely adhere to prescribing policies | ○ | ○ | ○ | ○ | ○ | ○ | ○ | ○ |
| 03. The development of shared care protocols | ○ | ○ | ○ | ○ | ○ | ○ | ○ | ○ |
| 04. Not prescribing "for QOF" if the medication is not actually needed (e.g. statin) | ○ | ○ | ○ | ○ | ○ | ○ | ○ | ○ |
| 05. Reduce unnecessary medication/antibiotic prescribing | ○ | ○ | ○ | ○ | ○ | ○ | ○ | ○ |
| 06. Allow the drug budgets to be able to purchase drugs with convenient dosage | ○ | ○ | ○ | ○ | ○ | ○ | ○ | ○ |
| 07. To give clear guidelines to patients as to how frequently they need medication reviews | ○ | ○ | ○ | ○ | ○ | ○ | ○ | ○ |
| 08. Reduce the work pressure to achieve targets | ○ | ○ | ○ | ○ | ○ | ○ | ○ | ○ |
| 09. Improve the GPs’ knowledge of drugs | ○ | ○ | ○ | ○ | ○ | ○ | ○ | ○ |
| 10. Educate patients on side effects, drug interactions and monitoring | ○ | ○ | ○ | ○ | ○ | ○ | ○ | ○ |
| 11. Improve patient information leaflets | ○ | ○ | ○ | ○ | ○ | ○ | ○ | ○ |
| 12. Provide more support for vulnerable elderly patients who have memory problems | ○ | ○ | ○ | ○ | ○ | ○ | ○ | ○ |
| 13. Alerts to appear on the computer screen also when re-issuing repeats and not just for first time prescribing | ○ | ○ | ○ | ○ | ○ | ○ | ○ | ○ |
| 14. Unified medication and investigations records that have listings of allergies and current medications of patients across primary and secondary care. This will allow GPs to see who changed, why, when and what medication or a result of a test ordered in secondary care | ○ | ○ | ○ | ○ | ○ | ○ | ○ | ○ |
| 15.  Closer and increased monitoring of toxic drugs | ○ | ○ | ○ | ○ | ○ | ○ | ○ | ○ |
| 16. Design the prescribing system so that certain medication such as pain-killers cannot be on list of repeat medications | ○ | ○ | ○ | ○ | ○ | ○ | ○ | ○ |
| 17. To increase the number of combination tablets | ○ | ○ | ○ | ○ | ○ | ○ | ○ | ○ |
| 18. Expand the use of aids that support adherence, e.g.  dosette boxes, alarm reminders, blister packs… | ○ | ○ | ○ | ○ | ○ | ○ | ○ | ○ |
| 19. Increase the use of e-referrals | ○ | ○ | ○ | ○ | ○ | ○ | ○ | ○ |
| Suggested solutions that could reduce or prevent medication-related patient safety problems in general practice | This solution is cost-effective | The implementation of this solution is feasible |
| Y | N | UnS | UnA | Y | N | UnS | UnA |
| 20. Increase the use of e-discharge letters | ○ | ○ | ○ | ○ | ○ | ○ | ○ | ○ |
| 21. Computer system to automatically inform the patient and the GP when blood tests are overdue | ○ | ○ | ○ | ○ | ○ | ○ | ○ | ○ |
| 22. Increased post-marketing surveillance on the effects of new and not so new medications | ○ | ○ | ○ | ○ | ○ | ○ | ○ | ○ |
| 23. All patients to have a named doctor | ○ | ○ | ○ | ○ | ○ | ○ | ○ | ○ |
| 24. Pharmacist to carry out medication reviews | ○ | ○ | ○ | ○ | ○ | ○ | ○ | ○ |
| 25. Labelling of medications by pharmacists should be in the patient’s first/best understood language | ○ | ○ | ○ | ○ | ○ | ○ | ○ | ○ |
| 26. To write indications next to each prescribed medication | ○ | ○ | ○ | ○ | ○ | ○ | ○ | ○ |
| 27. Develop standardized template for discharge summaries (e.g. with clear indications of changes from admission to discharge and with rationale) | ○ | ○ | ○ | ○ | ○ | ○ | ○ | ○ |
| 28. Greater use of near-patient INR testing in practices so that dosing and prescribing are integrated | ○ | ○ | ○ | ○ | ○ | ○ | ○ | ○ |
| 29. GP practices to follow stricter policies and procedures | ○ | ○ | ○ | ○ | ○ | ○ | ○ | ○ |
| 30. Allocate specific sessions and time slots for repeat prescribing (rather than fitting this task into the lunch break) | ○ | ○ | ○ | ○ | ○ | ○ | ○ | ○ |
| 31. To have face-to-face medication reviews as part of QOF | ○ | ○ | ○ | ○ | ○ | ○ | ○ | ○ |
| 32. To have more trained staff who can explain to patients why, how and when to take medication | ○ | ○ | ○ | ○ | ○ | ○ | ○ | ○ |
| 33. Carers should be allowed to administer medication | ○ | ○ | ○ | ○ | ○ | ○ | ○ | ○ |
| 34. Have community healthcare assistants who can go into patient’s homes to administer medication when compliance critical | ○ | ○ | ○ | ○ | ○ | ○ | ○ | ○ |
| 35. Regular medication reviews with focus on minimizing the number of medications and optimizing the dose | ○ | ○ | ○ | ○ | ○ | ○ | ○ | ○ |
| 36. Clear management plan which includes agreed 'aggressiveness' of therapy | ○ | ○ | ○ | ○ | ○ | ○ | ○ | ○ |
| 37. Greater emphasis on simplifying the treatment regimens e.g. use of once daily dosing | ○ | ○ | ○ | ○ | ○ | ○ | ○ | ○ |
| 38. Large print labelling with colour coding including patient information leaflets | ○ | ○ | ○ | ○ | ○ | ○ | ○ | ○ |
| 39. More time to review medication | ○ | ○ | ○ | ○ | ○ | ○ | ○ | ○ |
| 40. Systematic use of risk assessment tools | ○ | ○ | ○ | ○ | ○ | ○ | ○ | ○ |
| Suggested solutions that could reduce or prevent medication-related patient safety problems in general practice | This solution is cost-effective | The implementation of this solution is feasible |
| Y | N | UnS | UnA | Y | N | UnS | UnA |
| 41. Take patient’s co-morbidities more carefully into consideration when prescribing medications | ○ | ○ | ○ | ○ | ○ | ○ | ○ | ○ |
| 42. Develop a patient held record with up to date information on their medication | ○ | ○ | ○ | ○ | ○ | ○ | ○ | ○ |
| 43. Minimize polypharmacy | ○ | ○ | ○ | ○ | ○ | ○ | ○ | ○ |
| 44. Have closer links with relatives e.g. email/contact they could easily use | ○ | ○ | ○ | ○ | ○ | ○ | ○ | ○ |
| 45. Pharmacies should offer a check-and-collect service in addition to dispensing to only supply what’s needed. Explanation: Patients often have cupboards full of old medications | ○ | ○ | ○ | ○ | ○ | ○ | ○ | ○ |
| Please add any further ideas or comments that you believe are important and should be included in this study. |
|  |  |

| Is there anything else for patient safety in general practice that you would like to share with us? |
| --- |
|  |  |
| Thank you very much for your time and effort. Your contribution will help improve patient safety in general practice. For more information and to learn more about the outcomes of the study, please contact Dr. Josip Car at [josip.car@imperial.ac.uk](mailto:josip.car@imperial.ac.uk) |
