# Supplementary material for: Identification of priorities for improvement of medication safety in primary care: a PRIORITIZE study
Source: BMC Fam Pract. 2016 Nov 16;17:160. doi: 10.1186/s12875-016-0552-6 (PMC5112691; doi:10.1186/s12875-016-0552-6)
Supplement: Supplementary file 3 [file 12875_2016_552_MOESM3_ESM.odt]

Supplementary information S3 Text. Framework used for analysis of Contributing Factors to medication safety in primary care (based on the London protocol)* 

Patient Factors: condition (complexity & seriousness), language and communication, personality and social factors

Task and Technology Factors: task design and clarity of structure, availability and use of protocols, availability and accuracy of test results, decision-making aids

Individual (staff) Factors: knowledge and skills, competence, physical and mental health

Team Factors: verbal communication, written communication, supervision and seeking help, team structure (congruence, consistency, leadership, etc.)

Work Environmental Factors: staffing levels and skills mix, workload and shift patterns design, availability and maintenance of equipment, administrative and managerial support, physical environment

Organizational and Management Factors: financial resources & constraints, organizational structure, policy, standards and goals, safety culture and priorities

Institutional Context Factors: economic and regulatory context, National Health Service executive, links with external organizations

*Vincent C: Patient Safety. 2nd edition. Wiley-Blackwell; 2010.
