# Supplementary material for: Identification of priorities for improvement of medication safety in primary care: a PRIORITIZE study
Source: BMC Fam Pract. 2016 Nov 16;17:160. doi: 10.1186/s12875-016-0552-6 (PMC5112691; doi:10.1186/s12875-016-0552-6)
Supplement: Supplementary file 4 [file 12875_2016_552_MOESM4_ESM.odt]

Supplementary information S4 Text. Characteristics of the respondents to the initial questionnaire 

- Total number of survey respondents: 113

- GPs: 85, 75.2%

- GP Trainees: 17, 15.04%

- Foundation doctors: 4, 3.54%

- Blank: 3, 2.65%

- Nurses: 1, 0.88%

- Specialist trainees in Public Health: 1, 0.88%

- Consultants: 1, 0.88%

- Pharmacists: 1, 0.88%
