# Supplementary material for: Identification of priorities for improvement of medication safety in primary care: a PRIORITIZE study
Source: BMC Fam Pract. 2016 Nov 16;17:160. doi: 10.1186/s12875-016-0552-6 (PMC5112691; doi:10.1186/s12875-016-0552-6)
Supplement: Supplementary file 5 [file 12875_2016_552_MOESM5_ESM.odt]

S5 Table. Ranking of all (48) medication-related problems from primary care clinicians’ perspective (AEA range: 0 to 1) 

AEA –average expert agreement; TPS – total priority score

| Identified medication related problems in primary care | Breakdown point in the medication process | Main contributory factor | TPS | AEA | Responsiveness to solution | Frequency | Economic impact | Severity | Inequity |
| --- | --- | --- | --- | --- | --- | --- | --- | --- | --- |
| Incomplete reconciliation of medication during patient ‘hand-overs’ such as admission to and discharge from hospital or A&E | Transfer of care | Individual staff | 1 | 0,83 | 4 | 3 | 6 | 7 | 7 |
| Incorrect or insufficient patient education about the use of their medication e.g. how to take bisphosphonate or an inhaler | Administering | Patient | 2 | 0,82 | 12 | 4 | 4 | 14 | 1 |
| Poor discharge summaries | Monitoring | Individual staff | 3 | 0,83 | 1 | 5 | 7 | 2 | 24 |
| Polypharmacy in the elderly | Monitoring | Patient | 4 | 0,78 | 31 | 2 | 1 | 1 | 15 |
| Patient’s inability to understand or remember information about the daily doses or time of administration | Administering | Patient | 5 | 0,74 | 33 | 6 | 13 | 10 | 3 |
| Repeat prescribing without proper review, leading to continued use of unnecessary or unsafe medications | Monitoring | Individual staff | 6 | 0,76 | 5 | 9 | 5 | 6 | 20 |
| Time pressures- leading to prescribing errors and extended medication review times | Prescribing | Work environment | 7 | 0,75 | 32 | 1 | 2 | 4 | 21 |
| Long-term prescribing of NSAIDs without reviewing if there is an ongoing need for it | Prescribing | Individual staff | 8 | 0,76 | 13 | 14 | 3 | 3 | 26 |
| Repeat prescribing of pain-killers including opiates without a regular review of true need or alternatives | Prescribing | Individual staff | 9 | 0,7 | 9 | 10 | 10 | 12 | 12 |
| Delays in receiving notes when patient changes the practice they are registered with | Transfer of care | Task design | 10 | 0,74 | 3 | 7 | 17 | 17 | 23 |
| Limited continuity of care i.e. numerous doctors involved in the care of the same patient can lead to poor knowledge of the patient and the wrong medication being prescribed to them | Monitoring | Task design | 11 | 0,7 | 44 | 8 | 9 | 8 | 11 |
| Lack of medication review in home bound patients | Monitoring | Task design | 12 | 0,66 | 21 | 16 | 14 | 13 | 14 |
| Overdose, underdose or taking the wrong medications due to memory issues leading to a deterioration of health status | Administering | Patient | 13 | 0,64 | 25 | 28 | 12 | 9 | 9 |
| Insufficient checking of blood tests of patients on medications that require regular monitoring e.g. methotrexate, sulphasalazine, isotretinoin, lithium, etc. | Monitoring | Task design | 14 | 0,68 | 7 | 37 | 16 | 15 | 10 |
| Patient’s inability to articulate or recognize medication side effects that need action | Monitoring | Patient | 15 | 0,64 | 37 | 29 | 24 | 21 | 4 |
| Patient falls after being given medication (e.g. tranquilizers) | Prescribing | - | 16 | 0,69 | 24 | 31 | 8 | 5 | 30 |
| Confusion in medication names leading to patient safety threats | Administering | Task design | 17 | 0,63 | 35 | 27 | 23 | 28 | 5 |
| Some medications being prescribed in dosette boxes and others are not | Prescribing | Task design | 18 | 0,59 | 19 | 11 | 30 | 24 | 19 |
| GP advised by a specialist or hospital to prescribe a new medication without being given guidance on how to monitor this medication | Prescribing | Team | 19 | 0,69 | 14 | 12 | 18 | 23 | 38 |
| Pharmacies ordering prescriptions for patients early and then omitting any changes | Dispensing | Task design | 20 | 0,61 | 10 | 18 | 26 | 25 | 25 |
| Incomplete coding of patient problems | Monitoring | Individual staff | 21 | 0,68 | 8 | 15 | 22 | 26 | 34 |
| Insufficient monitoring of INR in patients on Warfarin | Monitoring | Task design | 22 | 0,62 | 6 | 41 | 20 | 22 | 13 |
| The history of past patient safety threats and adverse reactions to medications (e.g. NSAIDs and allergies) are not recorded in the patient notes (coding issues) | Prescribing | Individual staff | 23 | 0,65 | 15 | 35 | 19 | 19 | 31 |
| Inappropriate prescribing of pain management medication leading to drowsiness, falls | Prescribing | Individual staff | 24 | 0,58 | 29 | 25 | 25 | 16 | 29 |
| Patient taking the wrong dose | Administering | Patient | 25 | 0,56 | 39 | 22 | 28 | 42 | 2 |
| Hypotension and postural hypotension from antihypertensive | Monitoring | - | 26 | 0,67 | 38 | 20 | 11 | 11 | 40 |
| Prescribing of benzodiazepines when other alternatives exist | Prescribing | Individual staff | 27 | 0,58 | 17 | 33 | 33 | 27 | 16 |
| Carers allowed only to prompt and not give medication | Administering | Organizational | 28 | 0,55 | 26 | 23 | 29 | 29 | 22 |
| Lack of clarity about the drugs that are to be tailed-off following a discharge (e.g. steroids) | Transfer of care | Team | 29 | 0,62 | 20 | 13 | 36 | 34 | 28 |
| Carers not recognising medication side effects | Monitoring | Patient education | 30 | 0,51 | 34 | 32 | 34 | 31 | 8 |
| Nationals of other countries keep the GP in their country of origin and ask for medication that has been prescribed abroad without full information regarding indication, dose, etc. This leads to the wrong medication prescribed inadvertently | Transfer of care | Organizational | 31 | 0,55 | 42 | 26 | 15 | 33 | 17 |
| Hypoglycaemia due to taking medicines for diabetes | Monitoring | - | 32 | 0,58 | 43 | 36 | 21 | 18 | 27 |
| Patient being issued repeat prescriptions for an ACE-inhibitor without regular checks of U+Es | Monitoring | Individual staff | 33 | 0,56 | 11 | 30 | 39 | 41 | 18 |
| Wrong dose calculations for children and patients with renal disease and in other special situations | Prescribing | Task design | 34 | 0,6 | 23 | 38 | 27 | 20 | 37 |
| Medication has not been taken by a patient due to cost | Dispensing | Organizational | 35 | 0,53 | 41 | 40 | 32 | 35 | 6 |
| Illegible specialist’s prescription | Prescribing | Individual staff | 36 | 0,66 | 2 | 19 | 37 | 43 | 44 |
| Prescribing of rare, unusual and new drugs that the GP is not skilled in their use | Prescribing | Individual staff | 37 | 0,58 | 22 | 34 | 31 | 30 | 41 |
| Switching between generics and branded medication e.g. for antiepileptic medication | Prescribing | Task design | 38 | 0,55 | 18 | 21 | 40 | 36 | 35 |
| Too many irrelevant pop-ups and warnings on the computer screen (e.g. for interactions) which lead to the relevant items being ignored | Prescribing | Task design | 39 | 0,65 | 16 | 24 | 38 | 32 | 45 |
| Clicking/selecting the wrong drug/dose in the drop-down menu | Prescribing | Individual staff | 40 | 0,55 | 28 | 39 | 35 | 37 | 42 |
| Inappropriate prescribing of antipsychotics due to lack of clarity around indications for people with dementia leading to a deterioration of patient’s health or increased confusion | Prescribing | Task design | 41 | 0,4 | 40 | 43 | 42 | 38 | 33 |
| Continued pressure to change to cheapest medications | Prescribing | Organizational | 42 | 0,58 | 36 | 17 | 46 | 46 | 36 |
| Prescribing teratogen medications during pregnancy | Prescribing | Individual staff | 43 | 0,5 | 30 | 48 | 41 | 40 | 32 |
| Computer problems: the patient file that is being opened is not that of the patient who is in the room at the time of the consultation. This can happen with EMIS Web | Prescribing | Task design | 44 | 0,43 | 27 | 42 | 45 | 44 | 47 |
| Treatment of anger, aggression and violence with medication which is not licenced for these indications in elderly leading to drowsiness or even coma/death | Prescribing | Individual staff | 45 | 0,43 | 45 | 47 | 44 | 39 | 39 |
| Hypocalcaemia in chronic renal failure | Monitoring | - | 46 | 0,4 | 46 | 46 | 43 | 45 | 43 |
| The “SystmOne” screen feels more prone to errors as it is very full, the alerts are less concise and the GP does not have to tick a box before issuing medication | Prescribing | Task design | 47 | 0,47 | 47 | 45 | 47 | 47 | 48 |
| The “SystmOne” repeat prescriptions feature can allow patients to potentially get too much medication | Prescribing | Task design | 48 | 0,53 | 48 | 44 | 48 | 48 | 46 |
