# Supplementary material for: Identification of priorities for improvement of medication safety in primary care: a PRIORITIZE study
Source: BMC Fam Pract. 2016 Nov 16;17:160. doi: 10.1186/s12875-016-0552-6 (PMC5112691; doi:10.1186/s12875-016-0552-6)
Supplement: Supplementary file 6 [file 12875_2016_552_MOESM6_ESM.odt]

Supplementary information S6 Table. Ranking of all (45) solutions to medication-related problems from primary care clinicians’ perspective (AEA range: 0 to 1)

AEA –average expert agreement; TPS – total priority score

| Identified solution for medication safety problems in primary care | Breakdown point in the medication process | Related defence barrier | TPS | AEA | Cost-effectiveness | Feasibility |
| --- | --- | --- | --- | --- | --- | --- |
| Develop standardised template for discharge summaries (e.g. with clear indications of changes from admission to discharge and with rationale) | Prescribing | Task design | 1 | 0,96 | 3 | 1 |
| Reduce unnecessary medication/antibiotic prescribing | Prescribing | Task design | 2 | 0,96 | 1 | 11 |
| Minimize polypharmacy | Administering | Task design | 3 | 0,91 | 2 | 14 |
| Take patient’s co-morbidities more carefully into consideration when prescribing medications | Prescribing | Individual staff | 4 | 0,92 | 4 | 6 |
| Computer system to automatically inform the patient and the GP when blood tests are overdue | Monitoring | Task design | 5 | 0,92 | 7 | 5 |
| To give clear guidelines to patients as to how frequently they need medication reviews | Communication with patient/carers | Patient | 6 | 0,89 | 5 | 9 |
| Increase the use of e-discharge letters | Prescribing | Task design | 7 | 0,85 | 9 | 4 |
| Improve patient information leaflets | Communication with patient/carers | Patient | 8 | 0,86 | 6 | 12 |
| Unified medication and investigations records that have listings of allergies and current medications of patients across primary and secondary care. This will allow GPs to see who changed, why, when and what medication or a result of a test ordered in secondary care | Transfer of care | Task design | 9 | 0,85 | 12 | 3 |
| The development of shared care protocols | Transfer of care | Task design | 10 | 0,88 | 11 | 8 |
| Pharmacies should offer a check-and-collect service in addition to dispensing to only supply what’s needed. Explanation: Patients often have cupboards full of old medications | Dispensing | Task design | 11 | 0,86 | 13 | 7 |
| To write indications next to each prescribed medication | Prescribing | Task design | 12 | 0,85 | 8 | 16 |
| Greater emphasis on simplifying the treatment regimens e.g. use of once daily dosing | Administering | Task design | 13 | 0,80 | 10 | 18 |
| Greater use of near-patient INR testing in practices so that dosing and prescribing are integrated | Monitoring | Task design | 14 | 0,80 | 15 | 13 |
| Have community healthcare assistants who can go into patient’s homes to administer medication when compliance critical | Administering | Task design | 15 | 0,80 | 28 | 2 |
| Large print labelling with colour coding including patient information leaflets | Dispensing | Task design | 16 | 0,78 | 19 | 15 |
| Regular medication reviews with focus on minimising the number of medications and optimising the dose | Monitoring | Task design | 17 | 0,79 | 18 | 17 |
| Expand the use of aids that support adherence, e.g.  dosette boxes, alarm reminders, blister packs… | Administering | Task design | 18 | 0,78 | 26 | 10 |
| Educate patients on side effects, drug interactions and monitoring | Communication with patient/carers | Individual staff | 19 | 0,78 | 21 | 20 |
| Not prescribing "for QOF" if the medication is not actually needed (e.g. statin) | Prescribing | Task design | 20 | 0,76 | 20 | 21 |
| Carers should be allowed to administer medication | Administering | Task design | 21 | 0,74 | 17 | 22 |
| Alerts to appear on the computer screen also when re-issuing repeats and not just for first time prescribing | Prescribing | Task design | 22 | 0,75 | 16 | 24 |
| Closely adhere to prescribing policies | Prescribing | Individual staff | 23 | 0,73 | 14 | 30 |
| Increase the use of e-referrals | Transfer of care | Task design | 24 | 0,73 | 23 | 26 |
| Closer and increased monitoring of toxic drugs | Monitoring | Task design | 25 | 0,70 | 22 | 25 |
| Improve the GPs’ knowledge of drugs | Prescribing & monitoring | Individual staff | 26 | 0,73 | 25 | 23 |
| Provide more support for vulnerable elderly patients who have memory problems | Monitoring | Task design | 27 | 0,72 | 27 | 27 |
| Clear management plan which includes agreed 'aggressiveness' of therapy | Prescribing | Task design | 28 | 0,66 | 29 | 29 |
| More time to review medication | Monitoring | Organizational | 29 | 0,69 | 34 | 19 |
| Develop a patient held record with up to date information on their medication | Monitoring | Task design | 30 | 0,69 | 24 | 34 |
| Allow the drug budgets to be able to purchase drugs with convenient dosage | Prescribing | Organizational | 31 | 0,65 | 31 | 28 |
| Reduce the work pressure to achieve targets | Prescribing | Organizational | 32 | 0,68 | 30 | 31 |
| Pharmacist to carry out medication reviews | Monitoring | Work environment | 33 | 0,68 | 32 | 32 |
| Labelling of medications by pharmacists should be in the patient’s first/best understood language | Dispensing | Task design | 34 | 0,60 | 33 | 35 |
| Systematic use of risk assessment tools | Prescribing | Task design | 35 | 0,54 | 35 | 36 |
| Increased post-marketing surveillance on the effects of new and not so new medications | Monitoring | Organizational | 36 | 0,51 | 36 | 39 |
| Allocate specific sessions and time slots for repeat prescribing (rather than fitting this task into the lunch break) | Prescribing | Organizational | 37 | 0,54 | 42 | 33 |
| To have more trained staff who can explain to patients why, how and when to take medication | Communication with patient/carers | Organizational | 38 | 0,51 | 40 | 37 |
| Continuously and systematically double-check medication changes | Prescribing | Individual staff | 39 | 0,51 | 38 | 38 |
| Have closer links with relatives e.g. email/contact they could easily use | Communication with patient/carers | Task design | 40 | 0,48 | 37 | 42 |
| All patients to have a named doctor | Prescribing & monitoring | Organizational | 41 | 0,50 | 39 | 41 |
| To have face-to-face medication reviews as part of QOF | Monitoring | Task design | 42 | 0,47 | 43 | 40 |
| Design the prescribing system so that certain medication such as pain-killers cannot be on list of repeat medications | Prescribing & monitoring | Task design | 43 | 0,45 | 41 | 43 |
| GP practices to follow stricter policies and procedures | Prescribing & monitoring | Team | 44 | 0,42 | 44 | 44 |
| To increase the number of combination tablets | Administering | Task design | 45 | 0,51 | 45 | 45 |
